# Effects of alkali and transition metal-doped TiO_2_ hole blocking layers on the perovskite solar cells obtained by a two-step sequential deposition method in air and under vacuum†

**DOI:** 10.1039/d0ra01532f

**Published:** 2020-04-01

**Authors:** U. Nwankwo, Siphelo Ngqoloda, Agnes C. Nkele, Christopher J. Arendse, Kenneth I. Ozoemena, A. B. C. Ekwealor, Rajan Jose, Malik Maaza, Fabian I. Ezema

**Affiliations:** Department of Physics and Astronomy, University of Nigeria Nsukka Nigeria fabian.ezema@unn.edu.ng; Department of Physics/Geology/Geophysics, Alex Ekwueme Federal University Ndufu-Alike Ikwo Nigeria; Nanosciences African Network (NANOAFNET), iThemba LABS-National Research Foundation 1 Old Faure Road, Somerset West 7129, P.O. Box 722 Somerset West Western Cape Province South Africa; UNESCO-UNISA Africa Chair in Nanosciences/Nanotechnology, College of Graduate Studies, University of South Africa (UNISA) Muckleneuk Ridge, P.O. Box 392 Pretoria South Africa; Department of Physics and Astronomy, University of the Western Cape Private Bag X17 Bellville 7535 South Africa; Molecular Sciences Institute, School of Chemistry, University of the Witwatersrand Private Bag 3, P O Wits Johannesburg 2050 South Africa; Nanostructured Renewable Energy Materials Laboratory, Faculty of Industrial Sciences and Technology, Universiti Malaysia Pahang 26300 Kuantan Pahang Malaysia

## Abstract

Planar perovskite solar cells (PPSCs) have received great attention in recent years due to their intriguing properties, which make them a good choice for photovoltaic applications. In this work, the effect of alkali and transition metal-doped TiO_2_ (cesium-doped TiO_2_ (Cs-TiO_2_) and yttrium-doped TiO_2_ (Y-TiO_2_)) compact layers on the optical, structural and the photovoltaic performance of the PPSCs have been investigated. The perovskite layer syntheses were carried out by depositing a lead iodide (PbI_2_) layer *via* spin-coating; converting PbI_2_ into methyl ammonium iodide (CH_3_NH_3_PbI_3_) by chemical vapor deposition (CVD) and spin-coating at 60 min and 60 s conversion times respectively. The as-deposited PPSCs were studied layer-by-layer using an X-ray diffractometer, scanning electron microscope, and UV-vis diffuse reflectance, transmittance and absorbance. The power conversion efficiency for stable processed perovskite solar cells were 3.61% and 12.89% for air and vacuum processed, respectively.

## Abstract 2

Planar perovskite solar cells (PPSCs) have received great attention in recent years due to their intriguing properties, which make them a good choice for photovoltaic applications.

## Graphical abstract

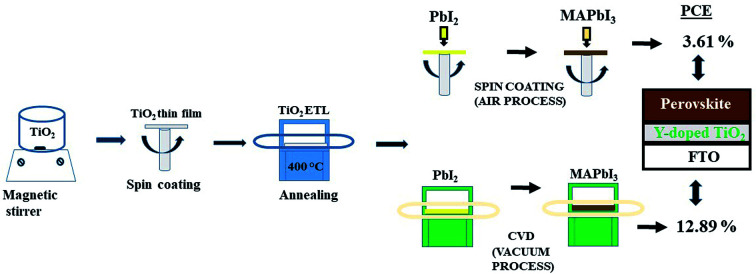

## 1. Introduction

Perovskite solar cells (PSCs) have attracted great interest due to their intriguing properties such as large absorption coefficient, high electron–hole diffusion length, tunable band gap, high charge carrier mobility, low temperature processing and low cost of production. These have made them good choices as efficient multi-purpose photovoltaics (PVs) for the next generation PV devices. The power conversion efficiency (PCE) of PSCs has been improved from 9.7%^1^ to 22.1%^2–4^ in the last seven years. The main device architectures of PCSs have been presented as planar and mesoporous, and are made up of the perovskite absorber layer, electron transport layer (ETL) and hole transport layer (HTL)^5–7^ on a fluorine-doped tin oxide (FTO) substrate. In the planar architecture, the perovskite absorber (CH_3_NH_3_PbI_3_) is usually sandwiched between ETL and HTL without a mesoporous layer while mesoporous architecture includes a mesoporous layer. Newly emerging device architectures are the inverted planar PSCs and planar structure PSCs without HTL.^8,9^ The perovskite absorber layer is the brain box of the perovskite photovoltaic devices where the charge carriers (excitons) are generated when light of energy greater than or equal to the optical bandgap of the absorber layer. Due to weak binding energy of the exciton (electron–hole), the separation of these electrons–holes take place at room temperature and requires an instant transport to ETL and HTL under the internal electric field at the junction.^10^ However, failure to transport the photo-generated charge carriers to the appropriate transport layer may result to quick recombination, charge accumulation at the interface and reduction in charge transfer.^11–13^ Due to the above mentioned reasons, efficient ETL is required to effectively extract the electron from the active layer before recombination occurs. A compact ETL, which is one component of the planar PSCs structure, has shown to be the most promising in producing a high and stable PCE. The planar structure without a mesoporous layer improves the perovskite crystallinity, surface morphology and reduce charge recombination.^14–16^

In the recent years, numerous metal oxides have been employed to improve the effectiveness of electron transport materials (ETM) such as titanium oxide (TiO_2_), aluminum oxide (Al_2_O_3_), zinc oxide (ZnO), tin oxide (SnO_2_), and magnesium oxide (MgO)^17–21^*etc.* TiO_2_ has been proven to be the most widely preferred ETM due to its chemical stability, low-cost synthesis and charge transport tendency.^10,22,23^ In addition, TiO_2_ ETM has shown a better conversion efficiency, and this is possible due to the band alignment between the conduction band of the TiO_2_ ETL and the lower unoccupied molecular orbital (LUMO) perovskite active layer.^24^ The electrons generated in the absorber layer can make his way to the ETL with less stress. Despite all these good names given to TiO_2_, it suffers from poor to low electrical conductivity. There is need to improve the electrical conductivity for better electron transport efficiency. One of the adopted ways to improve the electrical conductivity of the TiO_2_ ETL is metal ion doping. TiO_2_ metal ion doping have been reported in which several metal dopants such as lanthanum, lithium, niobium, aluminum and magnesium, were used for mesoporous doping on the TiO_2_ layer.^25,26^ The incorporation of these metal dopants resulted in tuning the Fermi level, reduced electronic trap sites, enhanced optical band gap and improved stability of the PSCs.^27–29^

Compact-TiO_2_ (c-TiO_2_) layer is the most frequently used ETL in the fabrication of highly efficient PSCs because of several reasons such as better exciton separation, easy to process in air, high transparency and low cost of fabrication.^30^ Varieties of deposition methods have been used in the preparation of c-TiO_2_ layer as ETL in PSCs, like spin-coating, dip-coating, spray-coating, magnetron sputtering, electrochemical deposition, electron beam deposition and atomic layer deposition.^31–34^ Spin-coating presents a lot of advantages over other deposition technique such as easy fabrication, low energy intensive, and thin film control through precursor concentration.^35^ The desired thickness of c-TiO_2_ layer is in the range of 10–80 nm for planar structure without mesoporous TiO_2_ layer. However, optimization of c-TiO_2_ layer for use as ETL in PSCs is currently an area of research that has received a great attention. The c-TiO_2_ layer is crucial by selectively allowing the electron generated at the absorber layer to be extracted to the electrode (FTO) so as to block the hole at the junction between FTO/absorber layer.^36,37^ It helps also to prevent carrier recombination at the perovskite/absorber interface, pinhole-free and maximum light penetration. Moreover, doped c-TiO_2_ layer have been reported to address the issue of hysteresis.^38^ More works are required to further improve the solar parameters and stability of planar (with no mesoporous layer) PSCs.

In this study, Cs and Y doped c-TiO_2_ ETL have been synthesized by sol–gel spin-coating deposition, which further promotes cost reduction of the PSCs. The planar architecture (n–i–p structure), FTO/doped c-TiO_2_/CH_3_NH_3_PbI_3_/HTL/Ag were used in all the fabrication steps. We further studied the crystal structure, electrical conductivity, optical properties, and layer-by-layer surface morphology of PSCs with the active layer deposited by two-step vapour deposition method. The PCE for perovskite layer deposited by spin coating and CVD methods was compared for different c-TiO_2_. The effects of the doped c-TiO_2_ and the active layer deposition technique on the performance of the fabricated PSCs were discussed in the article. Moreover, this research work addresses the issue of choice of deposition method for doped c-TiO_2_ ETL and the perovskite absorber layer on the PSCs performance.

## 2. Experimental

### 2.1 Materials

The materials used for this study were purchased as commercial products and some were used as purchased without any further purification. Methyl ammonium iodide (CH_3_NH_3_I), unpatterned FTO coated glass substrates (10 ohm per sq), 2,2′′,7,7′′-tetrakis(*N*,*N*-di-*p*-methoxyphenylamine)-9,9-spirobifluorene (Spiro-MeOTAD) were purchased at the Ossila. Lead(ii) iodide (PbI_2_; 99%), titanium isopropoxide (TTIP; 97%), chlorobenzene, 4-*tert*-butylpyridine (tBP), acetonitrile (99.8%), lithium bis-(trifluoromethanesulfonyl)imide (LITSFI), *N*,*N*-dimethylformamide (DMF; 99.8%), and dimethyl sulfoxide (DMSO; 99.9%) were purchased from Sigma and ALDRICH.

### 2.2 Preparation of c-TiO_2_ pristine and doped c-TiO_2_ layer

Prior to the deposition of c-TiO_2_ layer used in this study, the unpatterned FTO substrates were patterned by etching the desired portion with zinc (Zn) powder and 2 M hydrochloric acid (HCl). The etched substrates were cleaned in Hellmanex detergent, sonicated in deionized water (70 °C), isopropanol (IPA) and deionized water (70 °C) respectively. The cleaned FTO patterned substrates were treated by UV-O_3_ for 10 min.

The c-TiO_2_ layers were deposited on the patterned FTO substrates by sol–gel spin coating at 3000 rpm for 30 s, preheated for 10 min at 30 °C slightly above room temperature, and finally calcined at 400 °C for 1 h using hot plate. The titanium precursor solutions were prepared by adding different masses of cesium chloride (CsCl) and yttrium(iii) oxide (Y_2_O_3_) in ethanol (2.5 mL) and stirred for 2 h to dissolve completely. 350 μL of TTIP in ethanol (2.5 mL; 99.9%) in separate beaker was stirred for 10 min to form a milky solution. An acidic solution was prepared in a different beaker by adding 2 drops of HCl (2 M) in ethanol (2.5 mL). The dopant solution and acidic solution were added to the titanium precursor under constant stirring to clear the milky solution and form a viscous colorless solution. The prepared sol was spin-coated on the FTO patterned substrate by masking the undesired area for metal contact with acid resistive tape to form a thin FTO/c-TiO_2_. The same method was repeated for pristine c-TiO_2_ without adding any dopant oxides.

### 2.3 Fabrication of the perovskite absorber layer

**Fig. 1:**
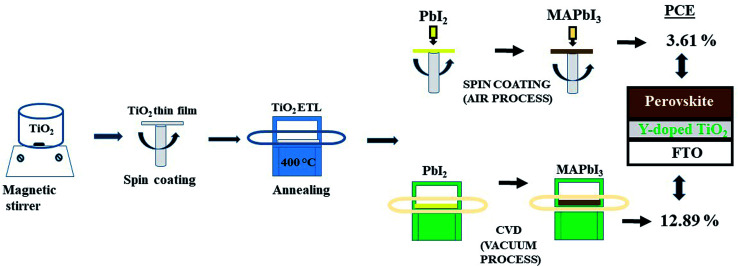
Schematic diagram of the deposition steps for the perovskite solar cell.

The perovskite absorber layer was prepared by two-step spin-coating and two-step chemical vapor deposition method. The schematic diagram of the deposition steps is shown in Fig. 1.

#### (I) Spin-coating deposition method

For the methyl ammonium lead iodide (MAPbI_3_) prepared by spin-coating deposition method, 369 mg of PbI_2_ was dissolved in a mixture of DMF and DMSO in the ratio of 9 : 1 respectively to form 0.8 M PbI_2_ solution. The PbI_2_ solution was heated at 65 °C for 3 h with the bottle containing the solution capped for the PbI_2_ to dissolve properly. With the PbI_2_ solution kept at 65 °C, 40 μL of the dissolved PbI_2_ solution was spin-coated on the FTO/c-TiO_2_ and FTO/c-TiO_2_ substrate which was heated at 65 °C prior to deposition at 7000 rpm for 35 s. The spin-coated PbI_2_ layer was preheated at 40 °C for 4 min, and finally heated at 105 °C for 4 min for the solvent to evaporate. This first procedure for the preparation of MAPbI_3_ by spin-coating is considered as the first step while the second step is to convert the PbI_2_ layer into MAPbI_3_. The conversion of the PbI_2_ into MAPbI_3_ was done by dissolving 10 mg of CH_3_NH_3_I in 1 mL of IPA, and then dropped on the PbI_2_ layer to diffuse into the layer for 60 s (loading time), followed by spin-coating at 4000 rpm for 20 s. The converted MAPbI_3_ was dried at 105 °C for 2 min to obtain a dark brown perovskite layer film. Afterwards, 50 μL of HTL (Spiro-OMeTAD) solution was spin coated on the MAPbI_3_ layer at 300 rpm for 30 s after which the perovskite layer was cooled to room temperature. The Spiro-OMeTAD solution was made from the mixture of 180 mg of 2,2′′,7,7′′-tetrakis(*N*,*N*-di-*p*-methoxyphenylamine)-9,9-spirobifluorene (Spiro-OMeTAD) in 1 mL of chlorobenzene, 30 μL of 4-*tert*-butylpyridine (tBP) and 20 μL of lithium bis-(trifluoromethanesulfonyl)imide (LITSFI) solution (520 mg of LITSFI in 1 mL of acetonitrile) stirred for 30 min and allow overnight to oxidize. Finally, 100 nm in thickness of silver (Ag) electrodes were deposited at a deposition rate of 0.1 Å s^−1^ using thermal evaporator to form a FTO/c-TiO_2_/MAPbI_3_/Spiro-OMeTAD/Ag device.

#### (II) Chemical vapor deposition method

The perovskite layer deposited by CVD was performed in two steps, whereby the PbI_2_ layer was deposited on the FTO/c-TiO_2_ and FTO/doped-TiO_2_ in the first step, followed by the conversion of the PbI_2_ layer to MAPbI_3_ in the presence of a MAI vapor. Further details of the deposition process can be found in the work by Ngqoloda *et al.*^39^

The procedures for HTL deposition layer and metal contact were repeated the same as the devices prepared by spin-coating technique to complete the device.

## 3. Characterization

The structural characterization of the synthesized TiO_2_ nanoparticles was carried out by an automatic powder X-ray diffractometer (XRD) X′ pert Pro with a theta–theta goniometer, using an ultrafast semiconductor detector pixel and Cu-Kα radiation (*λ* = 1.54 Å). The optical band gap, transmittance and absorbance were carried out with the Agilent Cary 5000 UV-VIS-NIR universal measurement spectrophotometer. The surface morphology and cross section imaging of the component layers were studied by Carl Zeiss mA 10 model field emission scanning electron microscopy (SEM) in combination with the energy dispersive X-ray spectroscopy (EDX) for elemental microanalysis. The current density–voltage (*J*–*V*) characteristics was measured with Keithley 2420 source meter under standard simulated solar irradiation of 1000 W m^−2^ (100 mW cm^−2^) and AM 1.5 at room temperature. The active area of the solar cell was 0.0512 cm^2^ as defined by the shadow mask used for the solar testing.

## 4. Results and discussion

### 4.1 Thickness optimization of Cs and Y doped TiO_2_ on FTO substrate

The desired thickness for the compact Cs-and Y-doped TiO_2_ layer was between 20 and 80 nm. The prepared solution of Cs-and Y-doped TiO_2_ was controlled by the spin speed, spin time, atmospheric temperature, stirring speed and time in order to achieve the thickness in the desired range. The deposition of the TiO_2_ layers were performed using 40 μL of the solution, spin speed of 3000 rpm and spin time of 30 s to achieve the desired average thickness of 54 ± 5 nm. Table 1 shows the summary of the obtained thicknesses with respect to spin-coated growth conditions.

**Table 1:** Thickness optimization with FTO substrates at different volumes, spin speeds and times

Amount of solution (μL)	Spin speed (rpm)	Spin duration (s)	Thickness (nm)
40.0	3000.0	30.0	48.0, 56.5 and 58.5
80.0	3000.0	30.0	130.0 and 142.0
40.0	2000.0	60.0	58.8, 71.0 and 53.0
100.0	3000.0	40.0	140.0 and 150.0
150.0	2000.0	60.0	2000.0

The above thicknesses were achieved at room temperature deposition using static spin-coating technique and measured with Dektak Profilometer.

### 4.2 XRD characterization

**Fig. 2:**
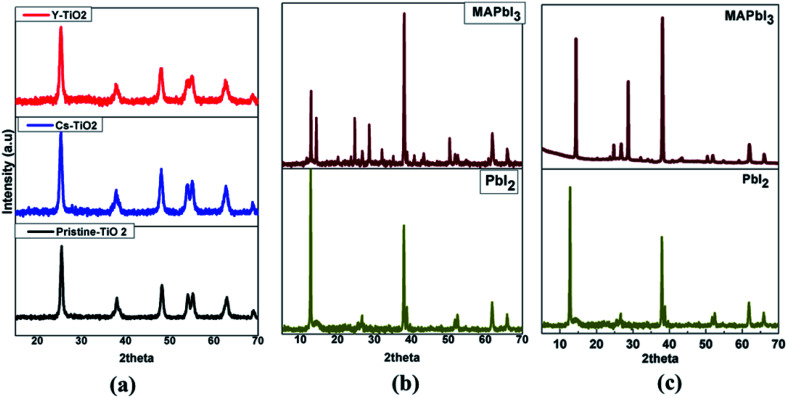
XRD Spectra of (a) pristine and doped TiO_2_ and (b) PbI_2_–MAPbI_3_ layer by spin-coating (c) PbI_2_–MAPbI_3_ layer by CVD.

Fig. 2 shows the XRD patterns for the pristine, doped Cs- and Y-TiO_2_ (Fig. 2(a)), perovskite layer processed in air (Fig. 2(b)) and vacuum (Fig. 2(c)).

The XRD patterns of the dried titanium precursor annealed at 500 °C for 30 min for the pristine, Cs- and Y-doped TiO_2_ show anatase phase of TiO_2_. The diffraction peaks observed at 2theta (indexed) values: 25.28° (101), 38.57° (112), 48.05° (200), 53.89° (105), 55.06° (211), 62.69° (204), and 68.76° (116) (as shown in Fig. 2(a)) indicates an anatase with body-centered tetragonal structure of TiO_2_ (JCP2-021-1272) with lattice parameters of *a* = *b* = 3.78520 Å, *c* = 9.51390 Å. The calculated lattice parameters for pristine TiO_2_, Cs-doped TiO_2_ and Y-doped TiO_2_ are (*a* = *b* = 3.7739 Å, *c* = 9.2928 Å), (*a* = *b* = 3.7858 Å, *c* = 9.5087 Å), and (*a* = *b* = 3.7898 Å, *c* = 9.4407 Å) respectively.

There is no other TiO_2_ phase observed in the XRD patterns of the samples which were all crystallized at 500 °C annealing.

The crystallite size of the pristine, Cs- and Y-doped TiO_2_ particles were estimated by the Scherer's equation;^40^1
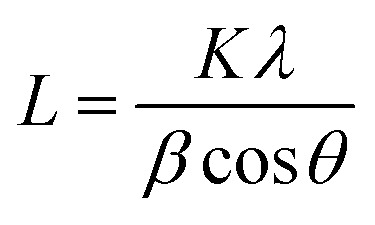
where *L* is the crystallite size, *K* is the Scherer constant (0.94 for FWHM of spherical crystals), *β* is the full width at half-maximum (FWHM) of the peak at 25.28° (101), *θ* is the diffraction angle and *λ* is the wavelength of X-ray source (Cu-Kα = 0.154 nm). The estimated crystallite sizes are 14.74, 14.07 and 13.07 nm for pristine TiO_2_, Cs-TiO_2_ and Y-TiO_2_ respectively using main diffraction peaks at 25.28° as shown in Fig. 2(a). There was a reduction in the crystallite size when doped with Cs and Y compare to the pristine TiO_2_. The decrease in crystallite sizes for Cs- and Y-doped TiO_2_ is expected as the ionic radius of both dopants play a role in the formation of the nanoparticles. The crystal with lesser crystallite size has smaller ionic radius.

The XRD pattern of the spin-coated PbI_2_ layer and converted MAPbI_3_ perovskite on the compact pristine, Cs- and Y-doped TiO_2_ layer are as shown in Fig. 2(b). The XRD spectra of the PbI_2_ layers show main peaks at 2theta diffraction angle of 12.72° corresponding to the reflection from (001) plane, with a hexagonal crystal structure (JCPDS 07-0235). The crystallite size was calculated using the Scherer eqn (1) and estimated to be approximately 42 ± 3 nm.

MAPbI_3_ was obtained from the conversion of PbI_2_ as shown in the XRD pattern in Fig. 2(b) [MAPbI_3_]. After the transformation process of PbI_2_ to MAPbI_3_ was completed, new sets of diffraction peaks (2theta) related to the tetragonal perovskite structure appeared at 14.20°, 19.95°, 28.79°, 31.88°, 40.72°, and 43.15° corresponding to (110), (112), (004), (312), (224), and (314) crystal planes respectively. The crystallite size using the 110 peak located at 14.20° was calculated to be 81 ± 3 nm and was larger than the size of PbI_2_ before conversion to MAPbI_3_. These MAPbI_3_ peaks are accompanied by other peaks from PbI_2_ and FTO layers, in which the PbI_2_ peak appearing at 12.72° was an indication that the conversion had some remnant PbI_2_ in the perovskite layer. There is no impurity peak from both the first and second step deposition of perovskite layer. The XRD patterns of the perovskite layers were the same for the perovskite layer on pristine, Cs- and Y-doped TiO_2_. However, the XRD pattern of the perovskite layer grown by CVD is shown in Fig. 2(c). The XRD pattern of the PbI_2_ layer grown by CVD is similar to the one grown by spin-coating with the main peak appeared at 12.70° diffraction angle corresponding to (001) Bragg reflection plane of the PbI_2_ crystal structure. The conversion of PbI_2_ to MAPbI_3_ results to a new set of major peaks at the 2theta diffraction position 14.04°, 24.40° and 28.37° corresponding to (110), (202) and (220) reflection planes. The XRD pattern of perovskite absorber layer was improved as no remnant PbI_2_ peak appear on the diffraction pattern. This show that the conversion from PbI_2_ to MAPbI_3_ for perovskite absorber layer grown by CVD was completely done, while for spin-coating some remnant PbI_2_ not converted was present in the MAPbI_3_ diffraction peaks.

### 4.3 SEM and EDX analyses of ETL, perovskite active and HTL layers

**Fig. 3:**
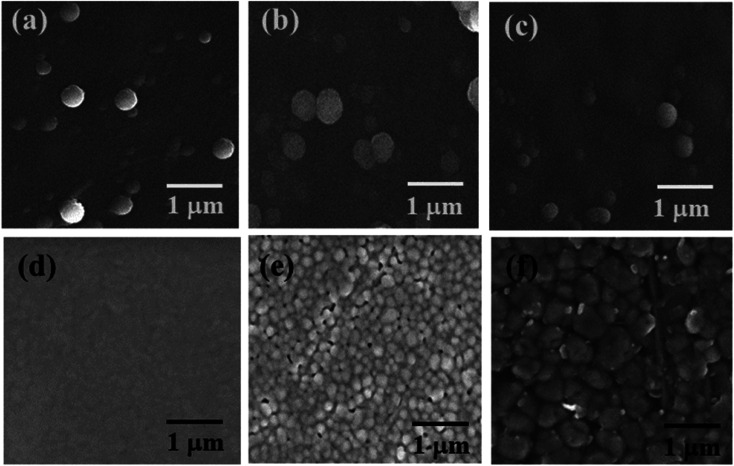
Top view SEM images of (a) pristine TiO_2_ compact layer (b) Cs-TiO_2_ compact layer (c) Y-TiO_2_ compact layer (d) PbI_2_ layer (e) MAPbI_3_ layer by spin-coating and (f) Spiro-OMeTAD layer.

**Fig. 4:**
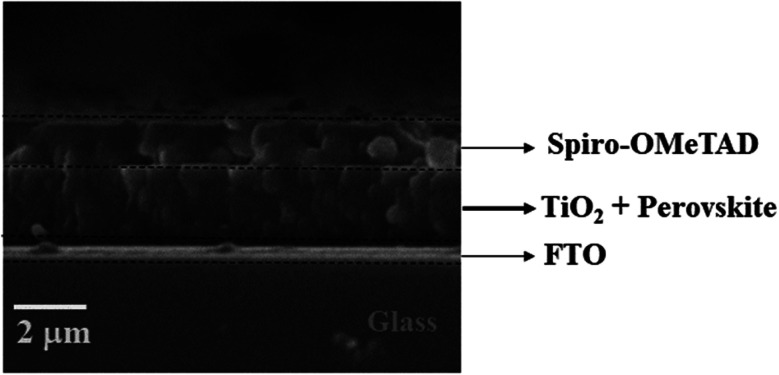
Cross-sectional SEM image of the TiO_2_ compact layer, perovskite layer and Spiro-OMeTAD layer.

The SEM micrograph of the top-view of the electron transporting layer (ETL), perovskite layer by spin-coating, hole transporting layer and perovskite layer by CVD are shown in Fig. 3(a–f) respectively and cross-sectional view in Fig. 4.

From Fig. 3(a–c); the SEM images are similar, and this is an indication that they particles grow in a similar manner. The dopant metal ions have no much effect on the surface morphology as shown in Fig. 3(b and c) compared with Fig. 3(a), which is the pristine TiO_2_. The SEM image for all the TiO_2_ layers had no cluster or agglomeration in the formation of the particle. The SEM image were spherical-like shaped in nature. The segregated nanoparticles were because the annealing temperature above 400 °C was able to initiate crystals which are similar in crystallite size as shown in Fig. 3(a–c).

The first step of the perovskite layer formation involves the deposition of densely packed PbI_2_ nanoparticles as shown in the SEM image of Fig. 3(d). The absence of pinhole in the deposition of PbI_2_ layer was as a result high spin speed and time during spin coating. The higher the spin speed and time, the better the surface morphology as seen in Fig. 3(d). The SEM image of the PbI_2_ layer appeared the same on the pristine, Cs- and Y-doped TiO_2_. However, the transformation of PbI_2_ layer to perovskite layer shows a different morphology with few pinholes and larger grain sizes in Fig. 3(e). The SEM image in Fig. 3(d and e) indicates that the grain size increases to almost double after conversion to perovskite layer in the second step of the perovskite deposition. The increase in grain size in this layer is advantageous because it prevents Spiro-OMETAD from diffusing into the ETL. This was also observed in the average crystallite size of the nanoparticles calculated with Scherer eqn (1) using the XRD spectra. However, the average grain size of the converted perovskite absorber layer deposited by spin coating technique was estimated from SEM image, and they are 0.43 ± 0.1, 0.22 ± 0.3, and 0.10 ± 0.1 μm for perovskite absorber layer deposited on Cs-doped TiO_2_, Y-doped TiO_2_ and pristine TiO_2_ respectively. The grain boundaries in the converted MAPbI_3_ layer increases during drying and thermal annealing of the perovskite layer to remove the remnant solvent. Fig. 3(f) shows the surface morphology of the deposited Spiro-OMeTAD layer with larger pores and grain size. The larger grain size was expected in this layer because the spin speed was reduced during spin coating. In addition, the SEM image of perovskite layer grown by CVD as depicted in Fig. S4† show larger grain size. The estimated average grain size of the perovskite layer grown by CVD is 0.857 ± 0.012 μm. From the SEM image in Fig. 3(e) and S4,† the grain boundaries of the perovskite layer grown by spin-coating method show some pin-holes, while the perovskite layer grown by CVD does not show any pin-holes in the grain boundaries. The absence of pin-holes in the perovskite layer grown by CVD is good as it prevents diffusion of HTL into the perovskite layer during deposition.

Fig. 4 shows the cross-sectional SEM image of the layers of the device from the FTO substrate to the Spiro-OMETAD in a sequential manner. The SEM image did not show interfacial diffusion in the layers of the perovskite solar cell. However, the perovskite layer thickness estimated to be larger than both the ETL and HTL was evident on the cross-sectional view of the three major layers in the solar cell device. The three layers created a well-defined interface in the SEM micrograph in Fig. 4. The cross-sectional SEM image of the perovskite layer grown by spin-coating is consistent with the cross-sectional SEM image of perovskite absorber layer grown by CVD as shown in our previous work.^39^

The EDX Spectra of the ETM, and the elemental composition confirmed that the dopant is present in the material (Fig. S1 ESI†). The EDX Spectra of the perovskite absorber layer show that the elemental compositions in the deposited materials are present (Fig. S2 ESI†). The absent of the dopant materials in the EDX of the perovskite absorber layer on the pristine, Cs-TiO_2_ and Y-TiO_2_ could be due to the small percentage amount of the dopant element in the ETL.

### 4.4 Optical transmittance and absorption spectra

The optical transmittance of the pristine, Cs- and Y-doped TiO_2_ deposited on FTO substrate is important prior to the deposition of the perovskite layer. This is to ascertain that reasonable amount of solar light can make the absorber layer (perovskite) to generate maximum photo-generated carriers for the solar performance.

Fig. S3,† the optical transmittance spectra for bare FTO substrate (black), pristine TiO_2_ (red), Cs-doped TiO_2_ (blue) and Y-doped TiO_2_ (green) measured in the UV-vis region of the electromagnetic spectrum. The optical transmittance spectra show an oscillating spectrum, due to interaction between the light and the atoms. The transmittance of the Cs-doped TiO_2_ on FTO substrate is lower compared to the other three (bare FTO, pristine TiO_2_ and Y-doped TiO_2_). The results are expected because the ionic radius of Cs dopant is much larger than ionic radius of Y dopant, and this tends to absorb more light. The transmittance for the three samples allowed over 50% light through them into the perovskite layer.

**Fig. 5:**
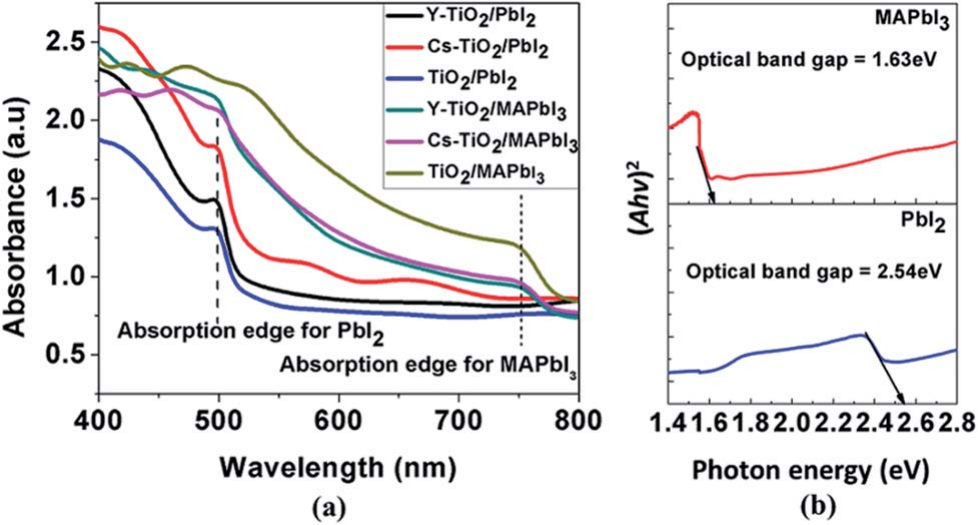
(a) Optical absorbance spectra of the perovskite on pristine, Cs doped and Y doped TiO_2_ and (b) the corresponding optical band gap of PbI_2_ and MAPbI_3_ calculated by Tauc plot.

Fig. 5 shows the optical absorbance spectra for the PbI_2_ before conversion to perovskite layer and MAPbI_3_ layer after conversion to perovskite layer. The absorbance was measured for PbI_2_ and MAPbI_3_ deposited on pristine, Cs-doped and Y-doped TiO_2_. From the absorbance spectra in Fig. 5(a), PbI_2_ deposited on the Cs-doped TiO_2_ showed the highest absorbance, followed by Y-doped TiO_2_.

The absorbance for PbI_2_ layers deposited on Cs- and Y-doped TiO_2_ are higher than PbI_2_ later deposited on pristine TiO_2_. This observation can be attributed to the larger ionic size of Cs dopant than Y dopant and pristine TiO_2_. However, the absorption edges in the PbI_2_ layers absorbance spectra are uniform and occur at 499 nm (2.49 eV). The calculated optical band gap of the PbI_2_ layer on the three different TiO_2_ substrates using the Tauc plot is approximately 2.54 eV. The optical band gap was calculated and extrapolated using Kubelka–Munk and Tauc plot for a direct band gap transition as shown in Fig. 5(b). Moreover, the absorption edge was shifted to the wavelength (750 nm) region upon conversion of PbI_2_ layer to MAPbI_3_ as shown in the Fig. 5(a). The calculated optical band gap of the converted MAPbI_3_ layer on the three different TiO_2_ substrates using Tauc plot is approximately 1.63 eV. The narrowing of the optical band gap of PbI_2_ layer transformed to MAPbI_3_ is as a result of structural modification caused by introducing MAI into the system. Both PbI_2_ and MAPbI_3_ thin film layers have higher absorption in the visible region of the electromagnetic spectrum.

### 4.5 *J*–*V* characteristics

**Fig. 6:**
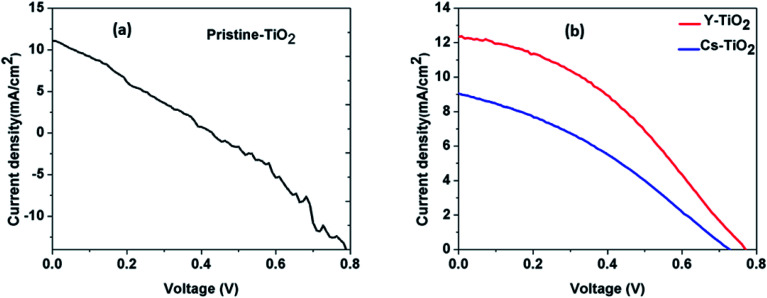
(a) Current density–voltage (*J*–*V*) curve for perovskite layer deposited on pristine TiO_2_ for air processed, (b) current density–voltage (*J*–*V*) curve for perovskite layer deposited on Cs-and Y-doped TiO_2_ for air processed.

**Fig. 7:**
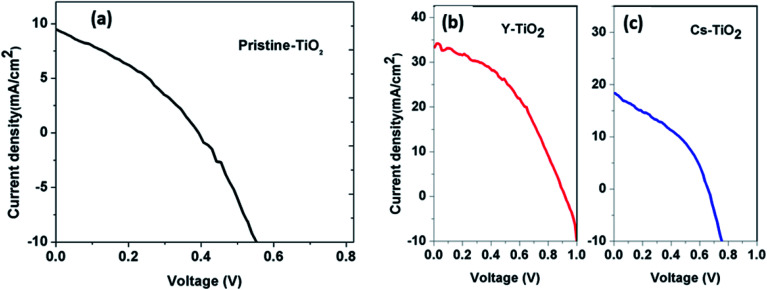
(a) Current density–voltage (*J*–*V*) curve for perovskite layer deposited on pristine TiO_2_ for vacuum processed, current density–voltage (*J*–*V*) curve for perovskite layer deposited on (b) Y- doped TiO_2_ and (c) Cs-doped TiO_2_ for vacuum processed.

Fig. 6 and 7 show the best performing solar cells fabricated with different perovskite deposition methods namely: (I) perovskite layer deposited by spin coating as air processed (II) perovskite layer deposited by CVD as vacuum processed. The key parameters *η* and FF of the perovskite solar cells were calculated using the eqn (2) and (3);^41^2
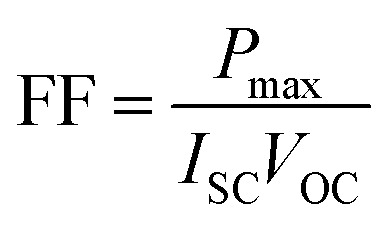
and3
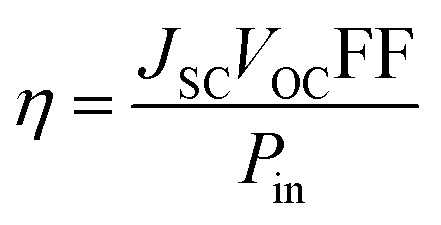
where *J*_SC_ is the current density (mA cm^−2^), *V*_OC_ is the open circuit voltage, FF is the fill factor and *P*_max_ is the maximum power.

#### (I) *J*–*V* characteristics of perovskite layer deposited by spin coating as air processed

From Fig. 6(a and b) and Table 2, we observed that power conversion efficiency (PCE) and solar parameter depends on the type of TiO_2_ layer in which perovskite layer is deposited upon. There is correlation between the average grain size and PCE of the device, which suggest that the lower the average grain size of the perovskite the better the PCE of device. The perovskite layer deposited on Y-doped TiO_2_ show the maximum PCE of 3.61%, and the lowest PCE of 1.45% was observed for perovskite layer deposited on pristine TiO_2_. The lower PCE observed for the perovskite layer on pristine TiO_2_ is due to lower value of the open circuit voltage, current density and fill factor. However, the nearly ohmic behavior observed in the *J*–*V* curve of the perovskite layer deposited on the pristine TiO_2_ may be due to the increase in the sheet resistance and series resistance of the fabricated solar cell.^41^

**Table 2:** Perovskite solar cell parameters for perovskite layer on different TiO_2_ by spin-coating and air processed

TiO_2_ layer	*J*_SC_ (mA cm^−2^)	*V*_OC_ (V)	FF	PCE (*η*) (%)
Pristine	10.71	0.44	0.31	1.45
Cs-doped	9.05	0.73	0.34	2.21
Y-doped	12.34	0.77	0.38	3.61

#### (II) *J*–*V* characteristics of perovskite layer deposited by CVD as vacuum processed

The obtained solar parameters from the *J*–*V* measurements of the best performing perovskite solar cells deposited on different ETL (pristine-TiO_2_, Cs-TiO_2_ and Y-TiO_2_ layer) are depicted in Fig. 7(a–c), and tabulated in Table 3. The solar cell with the high efficiency, fill factor and open circuit voltage out of the three different types of TiO_2_ is the TiO_2_ doped with yttrium. From Table 3, the solar parameters such as *J*_SC_, *V*_OC_ and FF increase with the PCE of the different TiO_2_ layer. After optimization process of the perovskite solar cell, a maximum efficiency of 12.89% was obtained for the perovskite layer deposited on Y-doped TiO_2_. This result indicates that after doping TiO_2_ with yttrium, the photovoltaic parameters can be improved. The doping of TiO_2_ is vital in the electron extraction from the active layer, and this was observed in the values of open circuit voltage for the three types of TiO_2_ layers. Depositing perovskite layer in a vacuum improved the *J*_SC_, *V*_OC_, FF and PCE of the perovskite solar cell as shown in Tables 2 and 3 The increase in the solar cell parameters is as a result of improved extraction of photo-generated charge carriers in the device. The increase in *V*_OC_ for the sample prepared by spin-coating and CVD can be attributed to the incorporation of Y and Cs dopant in the host element Ti. These dopants in the ETL alters the crystal structure of the pristine TiO_2_, and as a result, the Fermi-level shifted towards the conduction band edge of the TiO_2_. The closer the Fermi-level towards the conduction band edge, the better the extraction of electron to the bottom electrode. Perovskite absorber grown by both spin coating and CVD on doped Cs and Y-TiO_2_ show an increase in *V*_OC_ and *J*_SC_ which in turns improve the PCE. Consequently, *J*_SC_ usually increases when recombination rate is reduced in the photovoltaic devices, and we have reduced recombination rate in the perovskite layer by doping and by reducing the thickness of the electron transporting layer for effective electron extraction. However, the increase in *J*_SC_ of the device when perovskite layer is grown on compact Y-TiO_2_ and in other perovskite solar cell, have also been observed in the literature.^10,25,42,43^ High short-circuit current density of 28.06 mA cm^−2^ for perovskite solar cell has been reported,^42^ this value of *J*_SC_ is greater than the maximum predicted *J*_SC_ of 25 mA cm^−2^ for Pb based perovskite solar cell in the literature.^44^ The *I*–*V* characteristics of the complete devices deposited as perovskite absorber on Y-TiO_2_ by CVD show a higher *J*_SC_ of 32.60 mA cm^−2^, and these measurement was repeated for 4 different cells as depicted in Fig. S4–S7,† and all show similar high short-circuit current density above 25 mA cm^−2^. An enhanced PCE was observed for device processed in vacuum (CVD) than the device processed in air (spin-coating) due to the following reasons: (i) the exchange of oxygen in air processed perovskite devices which reduces the stability of the device during processing while there is no exchange of oxygen during vacuum processed, and (ii) the spin-coating processed perovskite devices have some pin-holes in the perovskite layer which could lead to the diffusion of HTL causing much recombination in the devices. In addition, the formation of voids sites in the pristine ETL could lead to reduction in extraction of photo-generated charge carrier. These voids sites may have been replaced by dopant in the host material to improve the PCE. The enhanced PCE has also been attributed to well-defined interface observed by sample grown by CVD than in spin-coating as reported in literature.^39,45^ Therefore, the method of deposition of the perovskite absorber layer determines the outcome of the PCE. This was observed in the perovskite absorber layer deposited by spin-coating with PCE 3.61% while the perovskite absorber layer deposited by CVD show a PCE of 12.89%.

**Table 3:** Perovskite solar cell parameters for perovskite layer on different TiO_2_ by CVD and vacuum processed

TiO_2_ layer	*J*_SC_ (mA cm^−2^)	*V*_OC_ (V)	FF	PCE (*η*) (%)
Pristine	9.88	0.39	0.35	1.35
Cs-doped	18.58	0.66	0.38	4.66
Y-doped	33.71	0.85	0.45	12.89

## 5. Conclusions

In summary, we successfully synthesized three different kinds of titanium precursors for the deposition of electron transporting layers to improve electron extraction from the perovskite active layer. The perovskite layer was deposited using two steps deposition by spin-coating in air and CVD in vacuum. The effect of doping TiO_2_ and the perovskite layer deposition method on the optical absorption, structural properties, surface morphology and efficiency were studied. The efficiency was enhanced by doping the TiO_2_ with yttrium which had ionic radius comparable with titanium(iv) ionic radius. However, short-circuit current density for complete devices made from perovskite absorber layer on Y-TiO_2_ for vacuum processed, show a higher *J*_SC_ above 25 mA cm^−2^. For perovskite layers deposited by spin coating and CVD, the efficiencies were calculated to be 3.61% and 12.89% respectively. These results suggest that a perovskite solar cells fabricated in a vacuum produce a high quality stable perovskite solar cell with an enhanced PCE.

## Conflicts of interest

There are no conflicts to declare.

## Supplementary Material

RA-010-D0RA01532F-s001
