# Supplementary material for: Effects of alkali and transition metal-doped TiO2 hole blocking layers on the perovskite solar cells obtained by a two-step sequential deposition method in air and under vacuum
Source: RSC Adv. 2020 Apr 1;10(22):13139–48. doi: 10.1039/d0ra01532f (PMC9051458; doi:10.1039/d0ra01532f)
Supplement: RA-010-D0RA01532F-s001 [file RA-010-D0RA01532F-s001.pdf]

Electronic Supplementary Material (ESI) for RSC Advances.
This journal is © The Royal Society of Chemistry 2020

SUPPORT INFORMATION

Effects of alkali and transition metal-doped TiO_2 hole blocking layers on the perovskite solar cells obtained by two-steps sequential deposition method in air and vacuum

U. Nwankwo[a,b,c,d,] , Siphelo Ngqoloda[e], Agnes C. Nkele[a], Christopher J. Arendse[e], Kenneth I. Ozoemena[f], A.B.C. Ekwealor[a], Rajan Jose[g], Malik Maaza[c,d], Fabian I. Ezema[a,c,d1*]

[a]*Department of Physics and Astronomy, University of Nigeria Nsukka Nigeria*

[b]*Department of Physics/Geology/Geophysics, Alex EkwuemeFederal UniversityNdufu-Alike,Ikwo, Nigeria*

[c] *Nanosciences African Network (NANOAFNET), iThemba LABS-National Research Foundation, 1 Old Faure road, Somerset West 7129, P.O.Box 722, Somerset West, Western Cape Province, South Africa*

[d]*UNESCO-UNISA Africa Chair in Nanosciences/Nanotechnology, College of Graduate Studies, University of South Africa (UNISA), Muckleneuk ridge, P.O. Box 392, Pretoria, South Africa*

[e] *Department of Physics and Astronomy, University of the Western Cape, Private Bag X17, Bellville, 7535, South Africa*

[f] *Molecular Sciences Institute, School of Chemistry, University of the Witwatersrand Private Bag 3, P O Wits, Johannesburg 2050, SOUTH AFRICA*

[g]*Nanostructured Renewable Energy Materials Laboratory, Faculty of Industrial Sciences and Technology, Universiti Malaysia Pahang, 26300 Kuantan, Pahang, Malaysia*

[*]Corresponding author: F.I. Ezema; fabian.ezema@unn.edu.ng

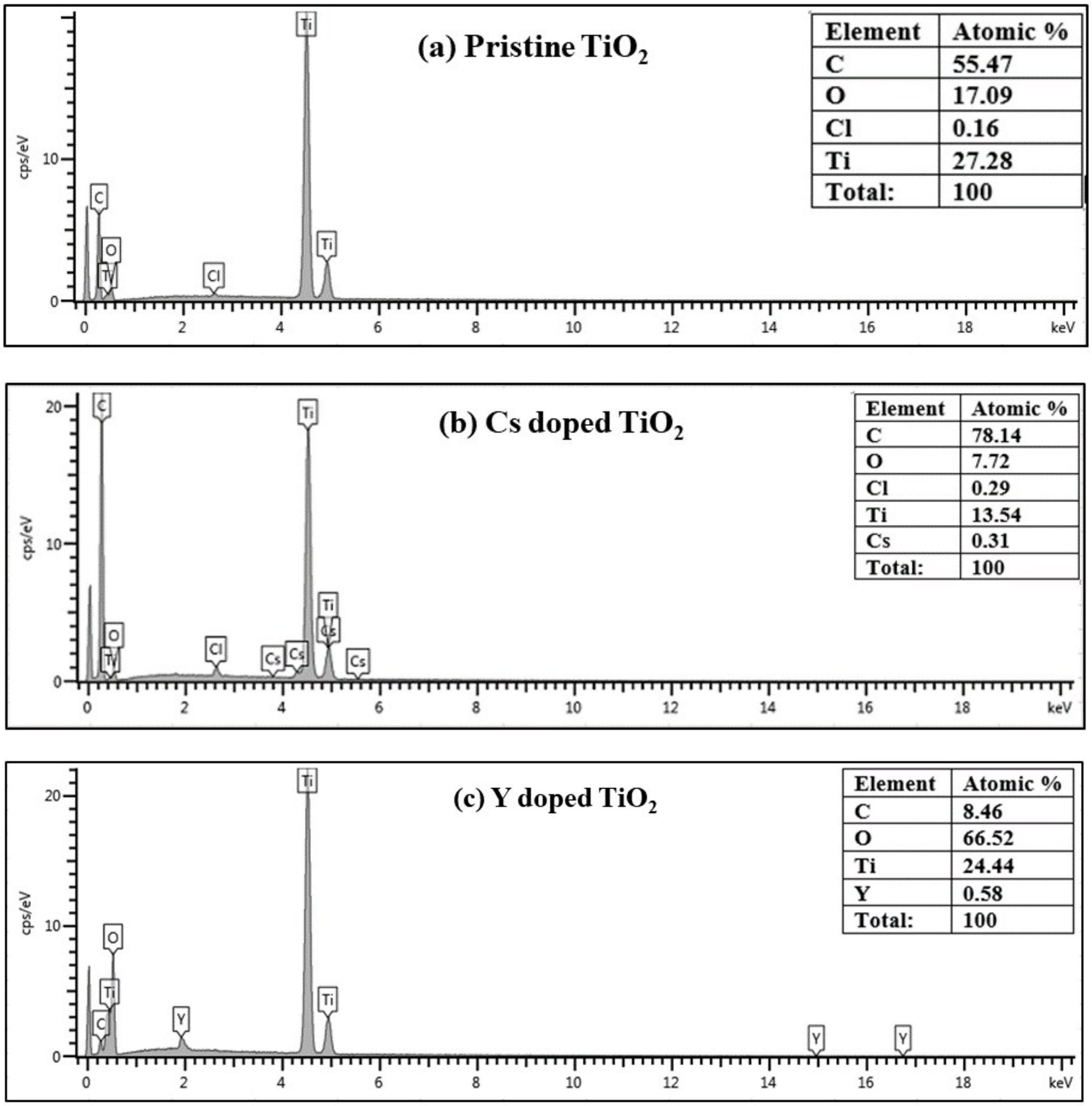

Figure S1: EDX Spectra of (a) Pristine TiO_2 (b) Cs-TiO_2 and (c) Y-TiO_2. The EDX measurements were performed along with the SEM imaging for the pristine, Cs- and Y-doped TiO_2 and the elemental composition of the dopant was confirmed in the material.

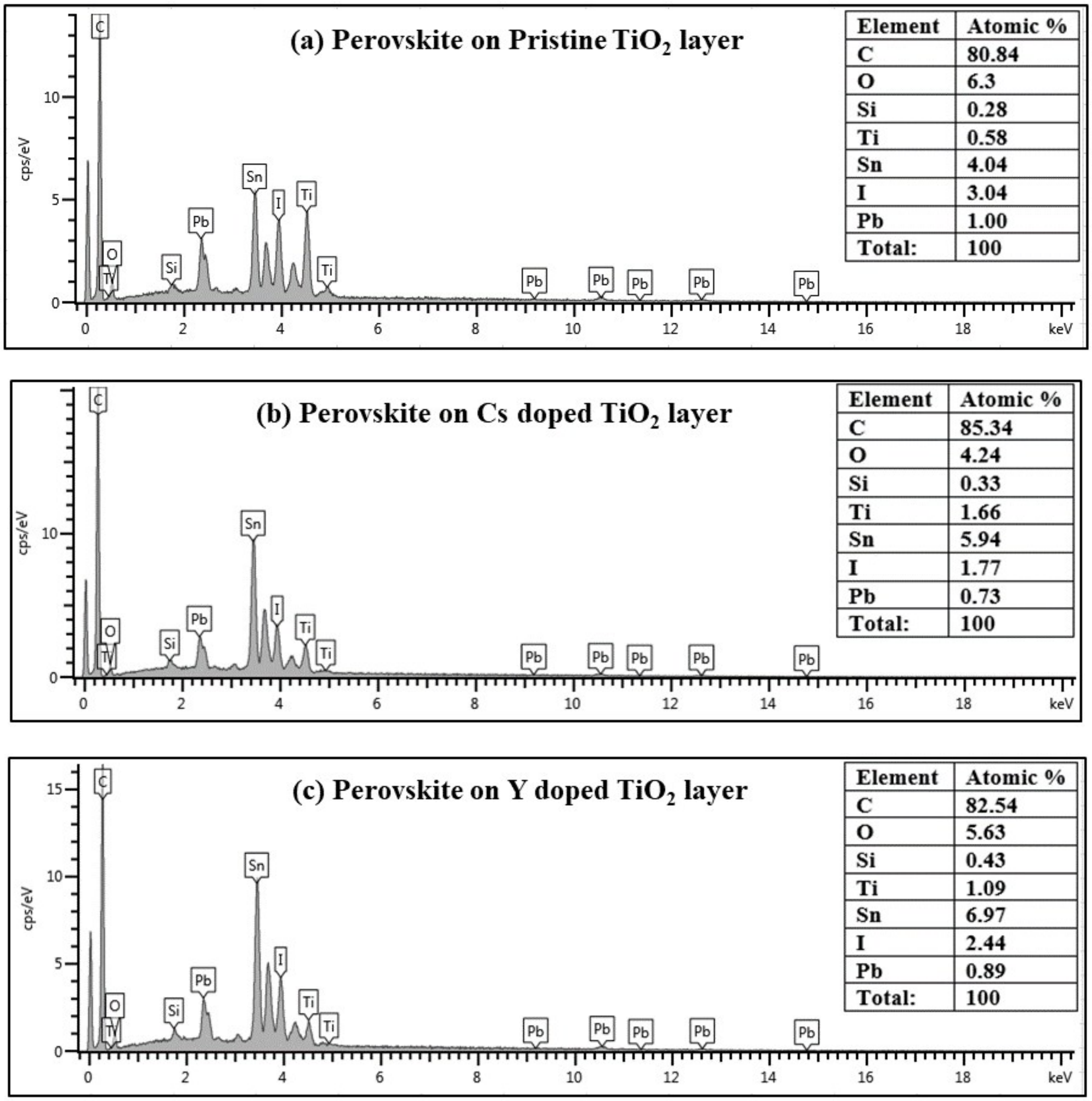

Figure S2: EDX Spectra of perovskite material on (a) Pristine TiO_2 (b) Cs-TiO_2 and (c) Y-TiO_2. The EDX measurement was performed along with the SEM imaging for the perovskite absorber layers grown on pristine, Cs- and Y-doped TiO_2 and the elemental composition of the dopant and perovskite material were confirmed in the spectrum.

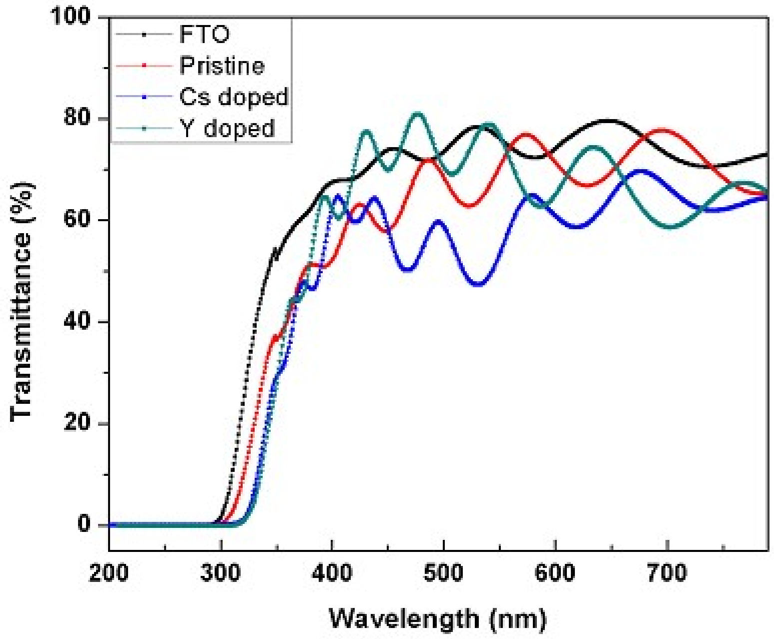

Figure S3: Optical transmittance spectra of the pristine, Cs-doped and Y-doped TiO_2. The optical transmittance spectra for Bare FTO substrate (black), Pristine TiO_2 (red), Cs-doped TiO_2 (blue) and Y-doped TiO_2 (green) measured in the UV-vis region of the electromagnetic spectrum prior to the deposition of the perovskite absorber layers.

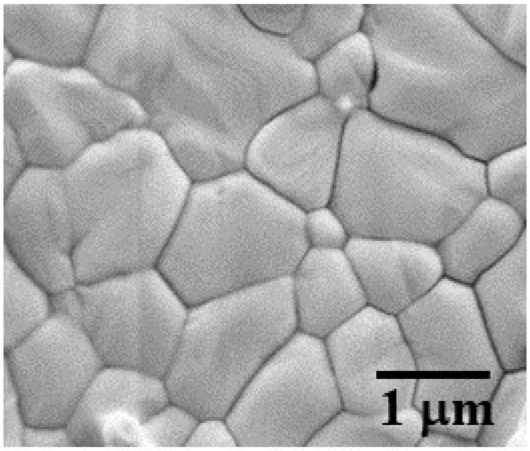

Figure S4: SEM image of Perovskite layer grown by CVD. The SEM image show a larger grain size compare to the perovskite layer grown by spin coating. The estimated average grain size of the perovskite layer grown by CVD is 0.857 ± 0.012 μm.

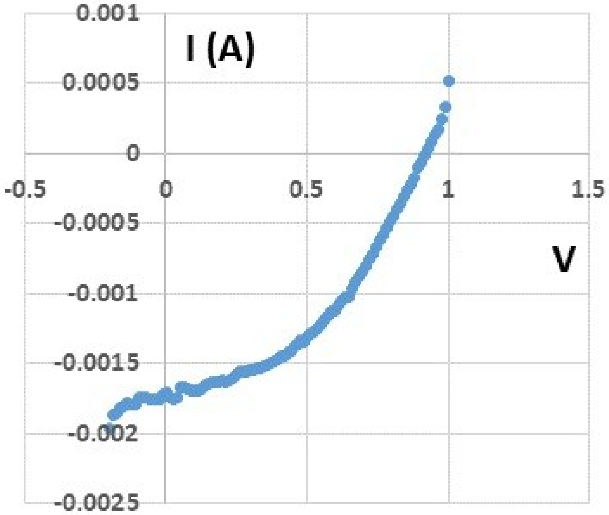

(a)

V_{oc} = 0.915V

I_{sc} = 0.0017A

I_{sc} = 1.7mA

Area of shadow mask = 0.0512 cm^2

J_{sc} = 33.20mA/cm^2

FF = 42%

(b)

Figure S5: (a) Current -voltage (I-V) curve for perovskite layer deposited on Y-doped TiO_2 for vacuum processed (b) Estimated perovskite solar cell photovoltaic parameters for perovskite absorber layer on Y-doped TiO_2 with high short-circuit current densities (J_{sc}) exceeding 25 mA/cm^2. This cell was measured along with the cell presented in the manuscript. The current-voltage (I-V) characteristics was measured with Keithley 2420 source meter under standard simulated solar irradiation of 100 mW/cm^2 and AM 1.5 at room temperature. The active area of the solar cell was 0.0512 cm^2 as defined by the shadow mask used for the solar testing.

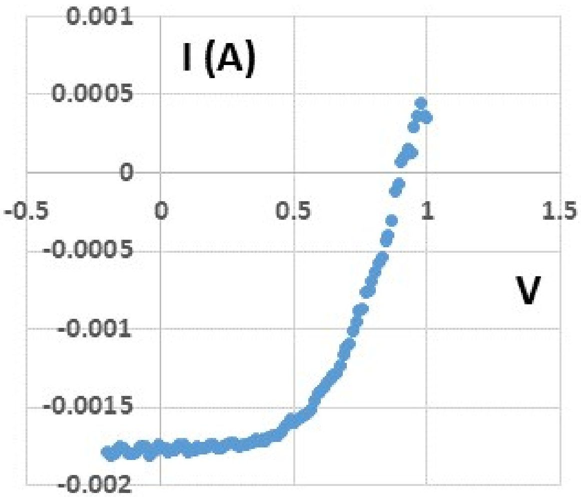

(a)

V_{oc} = 0.89 V

I_{sc} = 0.00176 A

I_{sc} = 1.76 mA

Area of shadow mask = 0.0512 cm^2

J_{sc} = 34.38 mA/cm^2

FF = 57%

(b)

Figure S6: (a) Current -voltage (I-V) curve for perovskite layer deposited on Y-doped TiO_2 for vacuum processed (b) Estimated perovskite solar cell photovoltaic parameters of another perovskite absorber layer grown on Y-TiO_2 layer. Measurements repeated on another to perovskite solar cell grown with the growth condition to compare with the short-circuit current density (J_{sc}) value presented in the main manuscript.

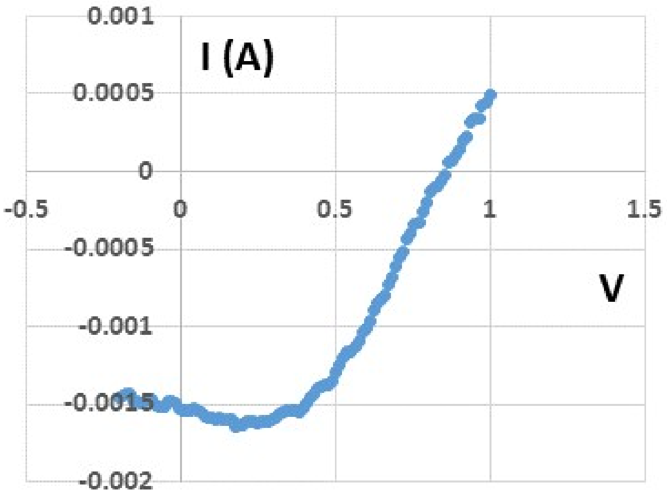

(a)

V_{oc} = 0.85 V

I_{sc} = 0.0015 A

I_{sc} = 1.7 mA

Area of shadow mask = 0.0512 cm^2

J_{sc} = 29.30 mA/cm^2

FF = 47%

(b)

Figure S7: (a) Current -voltage (I-V) curve for perovskite layer deposited on Y-doped TiO_2 for vacuum processed (b) Estimated perovskite solar cell photovoltaic parameters of another perovskite absorber layer grown on Y-TiO_2 layer.

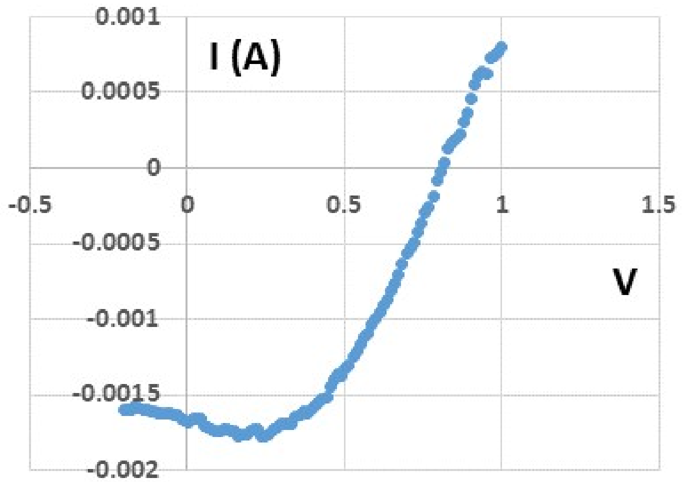

(a)

V_{oc} = 0.80 V

I_{sc} = 0.00166 A

I_{sc} = 1.66 mA

Area of shadow mask = 0.0512 cm^2

J_{sc} = 32.42 mA/cm^2

FF = 54%

(b)

Figure S8: (a) Current -voltage (I-V) curve for perovskite layer deposited on Y-doped TiO_2 for vacuum processed (b) Estimated perovskite solar cell photovoltaic parameters of another perovskite absorber layer grown on Y-TiO_2 layer. Measurements repeated on another to perovskite solar cell grown with the growth condition to compare with the short-circuit current density (J_{sc}) value presented in the main manuscript. Fig. S4 - S7 show that the short-circuit current density for all the perovskite absorber layer used in this work are high than 25 mA/cm^2.
